# Challenges in Paragangliomas and Pheochromocytomas: from Histology to Molecular Immunohistochemistry

**DOI:** 10.1007/s12022-021-09675-0

**Published:** 2021-03-25

**Authors:** C. Christofer Juhlin

**Affiliations:** 1 Department of Oncology-Pathology, Karolinska Institutet, Solna, Sweden; 2 Department of Pathology and Cytology, Karolinska University Hospital, Stockholm, Sweden

**Keywords:** Pheochromocytoma, Abdominal paraganglioma, Review, Histology, PASS, GAPP, Immunohistochemistry, Molecular diagnostics

## Abstract

Abdominal paragangliomas and pheochromocytomas (PPGLs) are rare neuroendocrine tumors of the infradiaphragmatic paraganglia and adrenal medulla, respectively. Although few pathologists outside of endocrine tertiary centers will ever diagnose such a lesion, the tumors are well known through the medical community—possible due to a combination of the sheer rarity, their often-spectacular presentation due to excess catecholamine secretion as well as their unrivaled coupling to constitutional susceptibility gene mutations and hereditary syndromes. All PPGLs are thought to harbor malignant potential, and therefore pose several challenges to the practicing pathologist. Specifically, a responsible diagnostician should recognize both the capacity and limitations of histological, immunohistochemical, and molecular algorithms to pinpoint high risk for future metastatic disease. This focused review aims to provide the surgical pathologist with a condensed update regarding the current strategies available in order to deliver an accurate prognostication of these enigmatic lesions.

## Introduction

Abdominal (sympathetic) paragangliomas and pheochromocytomas (PPGLs) are remarkable tumors, not only in terms of clinical presentation with a wide variety of symptoms derived from catecholamine production, but also with regards to the underlying tumor biology and its consequences for patient outcome. We now know that PPGLs constitute the most hereditable of all human tumors, with an established germline susceptibility event in approximately half of the patients [1–4]. Genetic events underlying the development of these lesions are noted in several different signaling pathways, of which mutations in genes regulating the tricarboxylic acid (TCA) cycle are particularly associated to metastatic disease [5]. Moreover, the former conception of “benign and “malignant” PPGL has now shifted towards a general appreciation that all tumors harbor malignant potential—thereby shifting the focus to a risk stratification approach to identify cases susceptible to future metastatic spread [1]. This focused review aims to cover the current histological, immunohistochemical and molecular approaches to pinpoint PPGLs at risk of dissemination, as well as to highlight the limitations. As parasympathetic paragangliomas of the head and neck region often are non-producing and clinically benign, they are not further discussed here [1].

### Epidemiology and Clinical Workup

Traditionally considered a “one in a million” disease, PPGLs have shown a rising incidence during the last 40 years, from 1.4 per million person-years in 1977 to 6.6 in 2015, constituting a 4.8-fold increase [6]. The upsurge is largely attributable to smaller tumors in patients with few/no symptoms, suggesting that an intensified use of clinical imaging techniques might contribute to this increase. Indeed, most PPGLs are diagnosed incidentally following radiological investigations rather than via symptoms of catecholamine excess [7, 8]. PPGLs are usually visualized on conventional CT or MRI scans, although functional modalities such as ^123^I-meta-iodobenzylguanidine (^123^I-MIBG) scintigraphy or ^68^ Ga-DOTATOC positron emission tomography (PET) often are needed to pinpoint the diagnosis [7, 9, 10]. In addition, plasma levels of chromogranin A and metanephrines are usually elevated [7, 11]. When localized to the primary site, cure rates are high if the tumor is surgically resected—however, treatment options for disseminated disease are limited [7, 12].

### Diagnostics

**Fig. 1:**
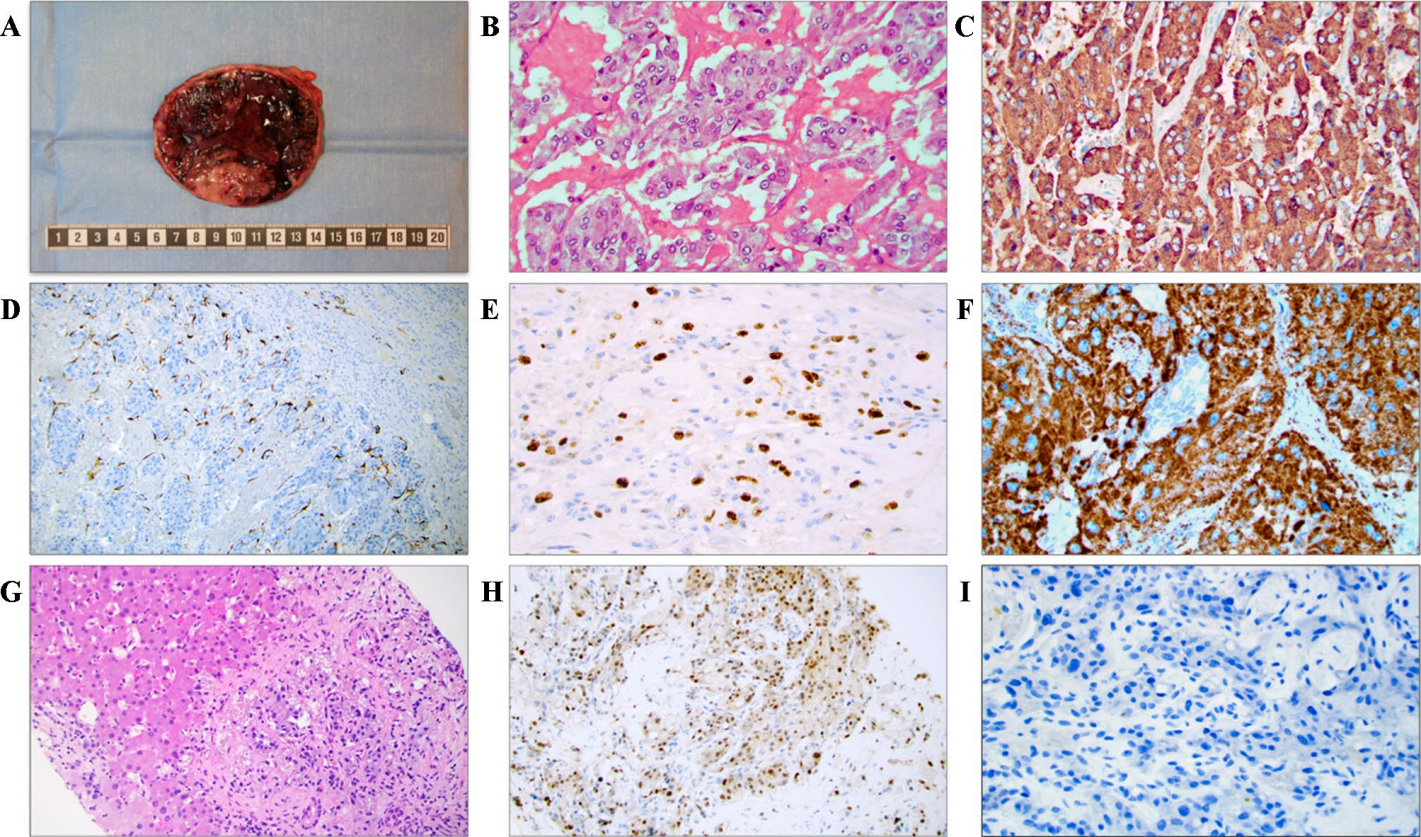
Key gross, microscopic and immunohistochemical findings of PPGLs. **A** Macroscopic appearance of the resected adrenal with a 10 cm encapsulated pheochromocytoma exhibiting a fleshy cut surface with solid tan-colored areas. **B** Photomicrograph of hematoxylin–eosin (H&E)-stained tumor tissue at × 400 magnification revealing a nested growth pattern and an exceedingly well-vascularized stroma. **C** Chromogranin A immunostaining at × 400 magnification. Note the diffusely positive cytosolic staining. **D** Sustentacular cells visualized using an S100 staining. **E** The Ki-67 proliferation index can be used to assess the proliferative activity, and is also a key part of certain algorithms to assess the metastatic potential. **F** Positive SDHB immunohistochemistry argues against underlying *SDHB*, *C*, and *D* gene mutations, thereby indicating a lower risk of disseminated disease. **G** Core needle biopsy of a liver metastasis in a patient previously resected for a pheochromocytoma 2 years earlier, with metastatic tumor cells recognizable through their basophilic cytoplasm. These tumor cells were positive for neuroendocrine markers (not shown). Upper left portion depicts hepatocytes. **H** GATA3 immunohistochemistry displaying nuclear positivity. **I** Complete loss of sustentacular cells was noted, as evident by an S100 immunostaining. This phenomenon is often reported in metastatic cases

As biopsies are generally not recommended for catecholamine-producing lesions, the diagnosis is typically made postoperatively after routine histopathological examination of the excised tumor [13]. Some baseline gross and microscopic characteristics are detailed in Fig. 1. Histologically, PPGLs often display a nested growth pattern (the so-called zellballen appearance) built-up by chief cells with abundant, basophilic cytoplasm. The tumor cells are usually surrounded by an arborizing network of thin blood vessels and supporting cells (“sustentacular cells”), which are recognizable only if immunohistochemical stains are applied (for example S100 and SOX10) (Fig. 1) [1]. The chief cells are strongly positive for neuroendocrine markers of both first (chromogranin A, synaptophysin) and second generation (ISL1, INSM1), and additional stains that may help distinguish PPGL include GATA3, tyrosine hydroxylase and dopamine beta-hydroxylase (Fig. 1) [1, 14–18]. Keratin expression is almost always absent, except for rare subtypes such as duodenal gangliocytic paragangliomas and cauda equina paragangliomas [14]. When assessing these lesions, the pathologist needs to consider many different aspects, including clinical information, primary tumor site, histology, the extent of invasion, the presence of vascular invasion, as well as the novel TNM staging system as dictated by the 8th edition of the American Joint Committee on Cancer (AJCC) Staging Manual [19, 20]. Interestingly, even though the TNM system for PPGL is newly adopted, the staging seems to reflect the biological properties and clinical outcomes when applied on retrospective materials [21]. In addition, the pathologist could also consider to implement histological scoring algorithms in order to stratify the future risk of metastatic disease [19]. Although all PPGLs are considered to exhibit malignant potential, only 10–15% of pheocromocytomas and between 30 and 50% of abdominal paragangliomas will metastasize to non-chromaffin sites [7]. Therefore, there is a considerable distinction between exhibiting malignant potential and actually exhibiting clinical features of malignancy (i.e., to metastasize). A metastatic pheochromocytoma is illustrated in Fig. 1.

### Underlying Genetic Aberrancies: with Focus on Metastatic PPGL

The genetics underlying the development of PPGLs is truly multifaceted. Following the identification of the *von Hippel Lindau* (*VHL)*, *Neurofibromatosis type (NF1)* and *Rearranged during transfection* (*RET*) genes responsible for the VHL, NF1 and the multiple endocrine neoplasia type 2 (MEN2) multitumor syndromes respectively in which PPGL is a recurrent feature [22–25], the list has expanded considerably with the advent of comprehensive next-generation sequencing techniques. To date, germline alterations in more than 20 genes have been associated to the development of PPGL, and for this reason, the current WHO classification recommends genetic screening of all PPGL patients [1, 26]. From a histological point of view, the endocrine pathologist should also assess whether or not synchronous adrenomedullary hyperplasia is present when diagnosing pheochromocytoma, as this feature may be a clue to the occurrence of predisposing gene mutations [27, 28]. Germline mutations are most commonly detected in the *Succinate dehydrogenase complex flavoprotein subunit B* (*SDHB*), *RET*, *VHL* and *NF1* genes, while somatic mutations are most commonly observed in *Harvey rat sarcoma viral oncogene homolog* (*HRAS*), *NF1*, *Endothelial PAS domain-containing protein 1* (*EPAS1)* and *RET* [3, 29–40]. Comprehensive genetic reviews regarding these genes and their associated *modus operandi* in terms of influencing PPGL development have been published elsewhere [2, 3, 5].

**Fig. 2:**
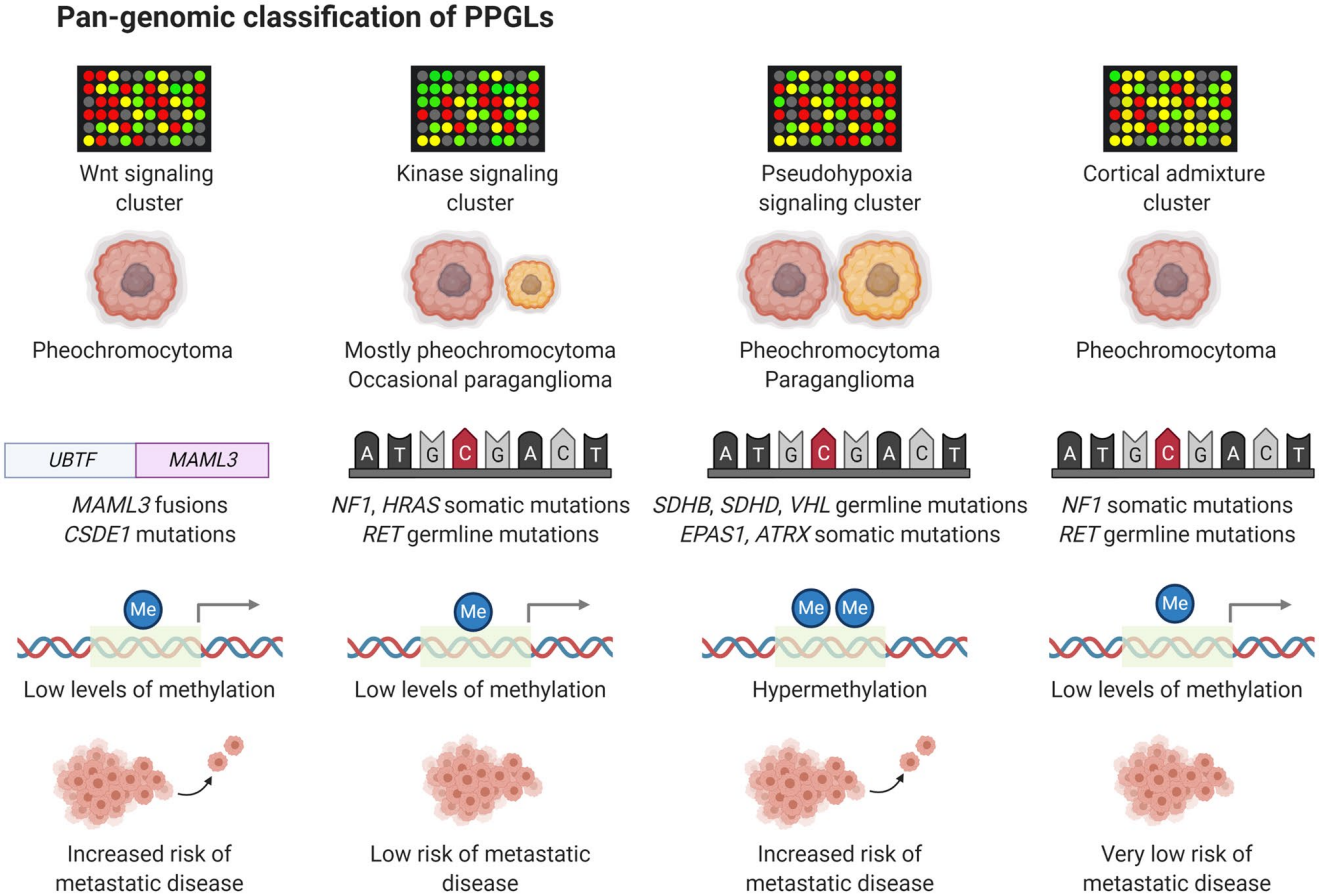
Pan-genomic classification of PPGLs. By in-depth transcriptome profiling, PPGLs generally adhere to one out of four principal expressional clusters, the Wnt pathway cluster, the kinase-associated cluster, the pseudohypoxia-associated cluster, and the cortical admixture cluster. The Wnt cluster is enriched for pheochromocytomas with somatic *MAML3* gene fusions as well as *CSDE1* gene mutations, and these tumors have an increased risk of metastasizing compared to kinase associated and cortical admixture cluster lesions. The kinase associated cluster is built-up primarily of pheochromocytomas with low metastatic potential. Tumors in this cluster exhibit mutations in kinase-associated pathways, and genes include *NF1*, *RET*, *HRAS*, *MAX*, *TMEM127*, and *KIF1B*. The pseudohypoxia cluster includes pheochromocytomas and paragangliomas with tricarboxylic acid (TCA) cycle mutations (*SDHx* gene family, multiple other TCA cycle related enzymes) or mutations in hypoxia-inducible factor (HIF)-associated signaling networks (*VHL*, *EPAS1/HIF2-α*, *PHD1/EGLN2*, and *PHD2/EGLN1*). This cluster contains a subgroup of cases (mostly TCA cycle aberrant) with global hypermethylation, which is a feature generally associated to metastatic disease and worse clinical outcomes. The fourth group (cortical admixture) is represented by pheochromocytomas with kinase-associated mutations and low risk of metastatic disease. In all, 24.3% of patients with PPGLs adhering to the pseudo-hypoxia cluster exhibit metastatic disease, compared to 11.4% of patients with Wnt cluster PPGLs and 4.1% of patients with PPGLs associated to the kinase cluster [5]. In contrast, patients with PPGLs associated to the cortical admixture cluster almost always demonstrate a benign clinical course [29]

**Fig. 3:**
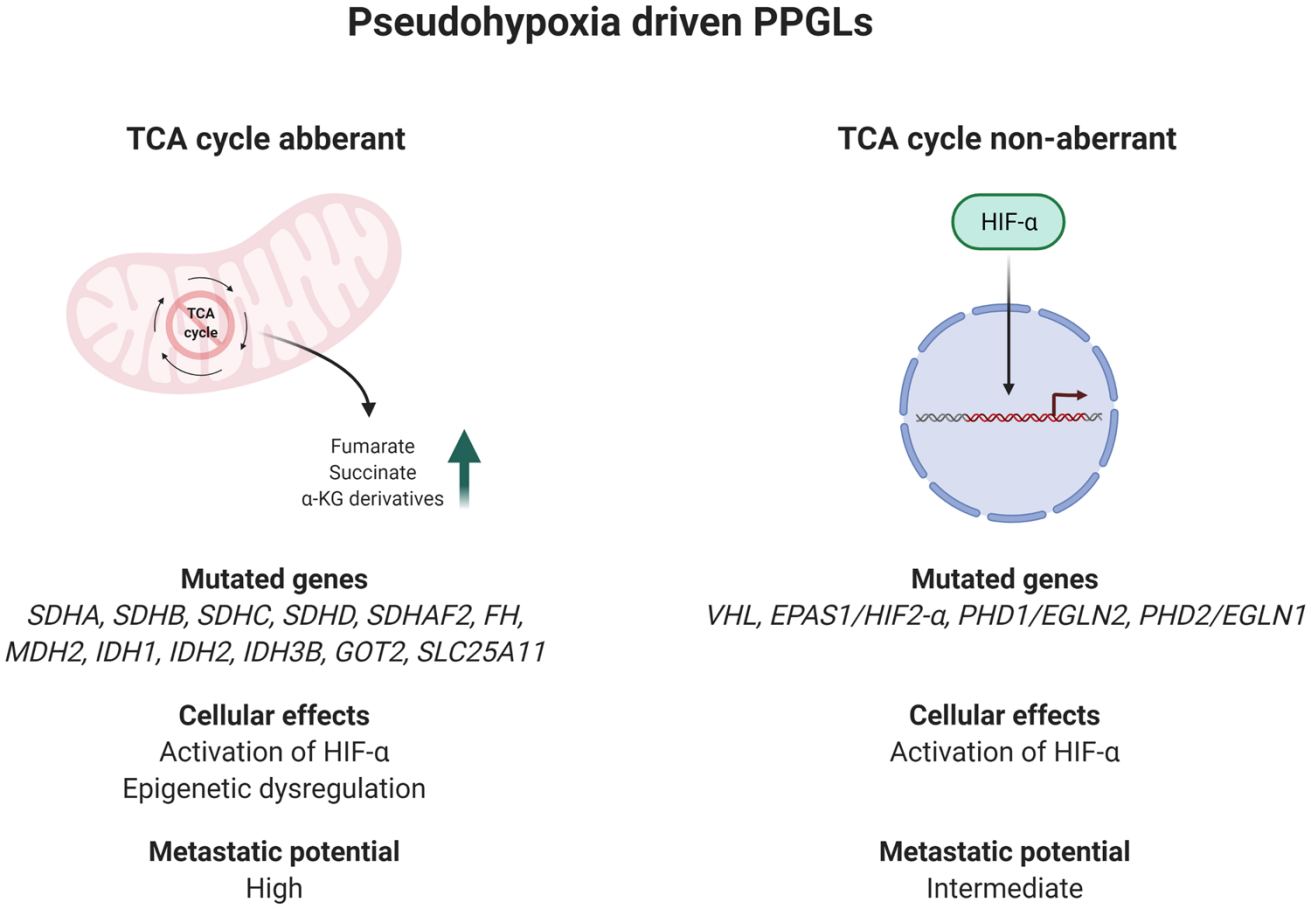
Pseudohypoxia-driven PPGLs. The pseudo-hypoxia cluster of PPGL is traditionally associated to worse clinical outcome, but a closer look at the sub-stratification of this cluster reveals an association between mutations in genes implicated in the regulation of the tricarboxylic acid (TCA) cycle and increased metastatic potential, as opposed to TCA cycle non-aberrant PPGLs within the same cluster. While both subgroups activate the HIF signaling pathway, TCA cycle mutated tumors also exhibit epigenetic dysregulation as a consequence of the accumulation of onco-metabolites (fumarate, succinate, and α-ketoglutarate (α-KG) derivatives. The risk of metastatic events among patients with pseudohypoxia-driven PPGLs is much higher in TCA cycle aberrant cases (40.5% of patients) than in TCA cycle non-aberrant cases (11.2% of patients) [5]

**Fig. 4:**
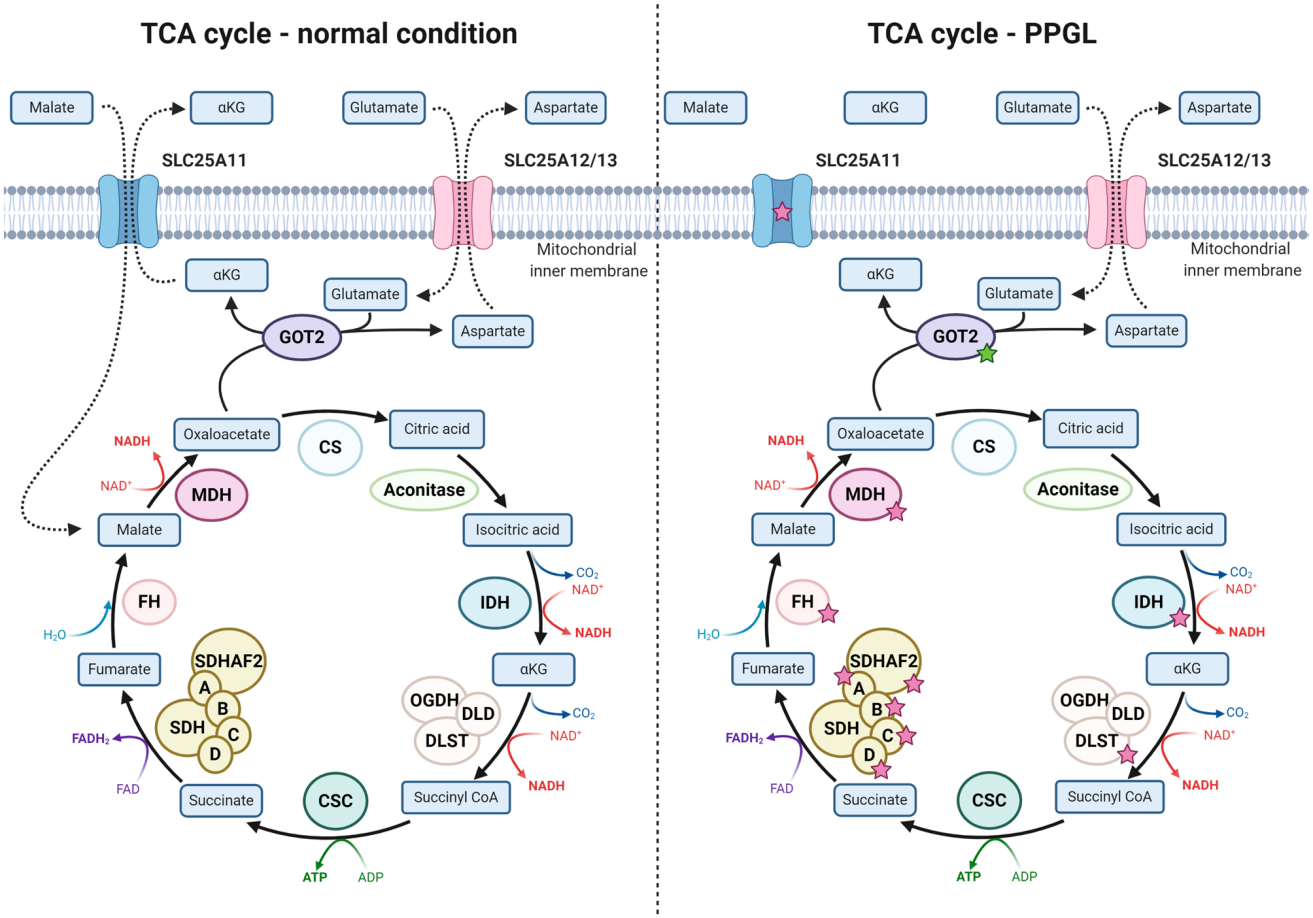
Tricarboxylic acid (TCA) cycle dysregulation in PPGLs. In normal circumstances, the TCA cycle is an orchestrated chain of events propelled by a number of key enzymes, in which energy is extracted through the oxidation of acetyl-CoA. Each step of the TCA cycle is tightly regulated by enzymes, of which the majority can be inactivated through gene mutations (red stars) in PPGLs. These events are thought to cause an accumulation of key metabolites, in turn affecting other cellular processes reviewed in upcoming figures. Mutations in *isocitrate dehydrogenase* (*IDH*), the α-ketoglutarate dehydrogenase subunit *dihydrolipoamide s-succinyltransferase* (*DLST*), *succinate dehydrogenase complex flavoprotein subunits A, B, C, D* and *succinate dehydrogenase complex assembly factor 2* (*SDHAF2*), *fumarate hydratase* (*FH*) and *malate dehydrogenase* (*MDH*) have all been reported on either the somatic or germline level in PPGL. Moreover, gene mutations involving transporter molecules responsible for the shuttling of malate and α-ketoglutarate (*SLC25A11*), as well as activating mutations (green star) in genes catalyzing the conversion of glutamate to α-ketoglutarate (*GOT2*), are also reported

**Fig. 5:**
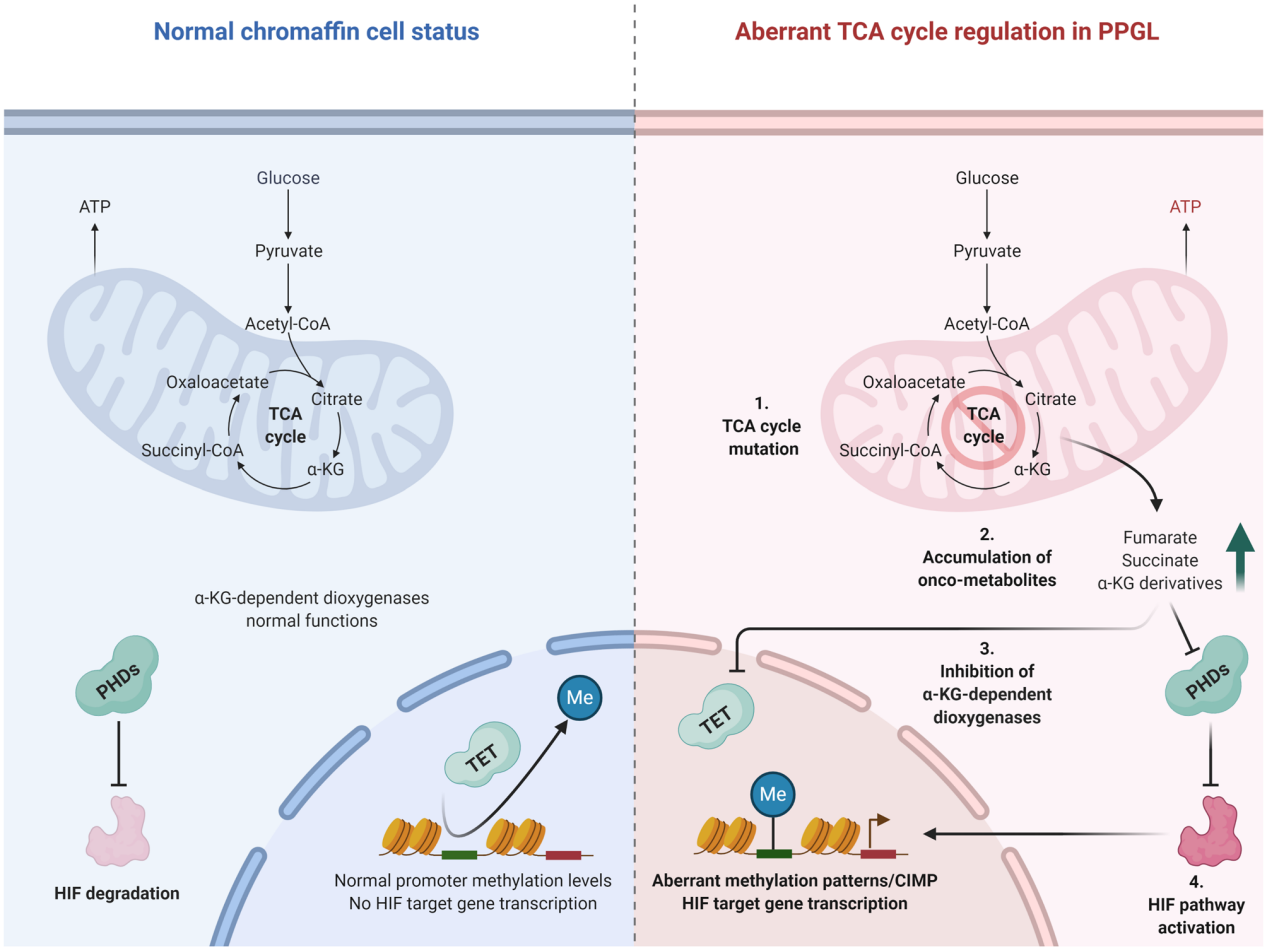
Cellular consequences of an aberrant tricarboxylic acid (TCA) cycle in PPGLs. In the normal cell state, the TCA cycle operates at normal capacity, generating ATP and keeping extra-mitochondrial levels of onco-metabolites (succinate, fumarate, and α-ketoglutarate (α-KG)) derivative levels low. Thus, the diverse group of α-KG dependent dioxygenase enzymes (including PHD enzymes targeting the HIF oncoprotein for degradation as well as TET proteins responsible for de-methylating the genome) is functional. Upon aberrant TCA cycle regulation (via mutations in genes encoding enzymes catalyzing key steps of the TCA cycle), accumulation of extra-mitochondrial succinate, fumarate and α-KG metabolites will inhibit dioxygenase function, leading to impaired PHD and TET enzyme activity—thereby promoting HIF pathway activation and genomic hypermethylation

**Fig. 6:**
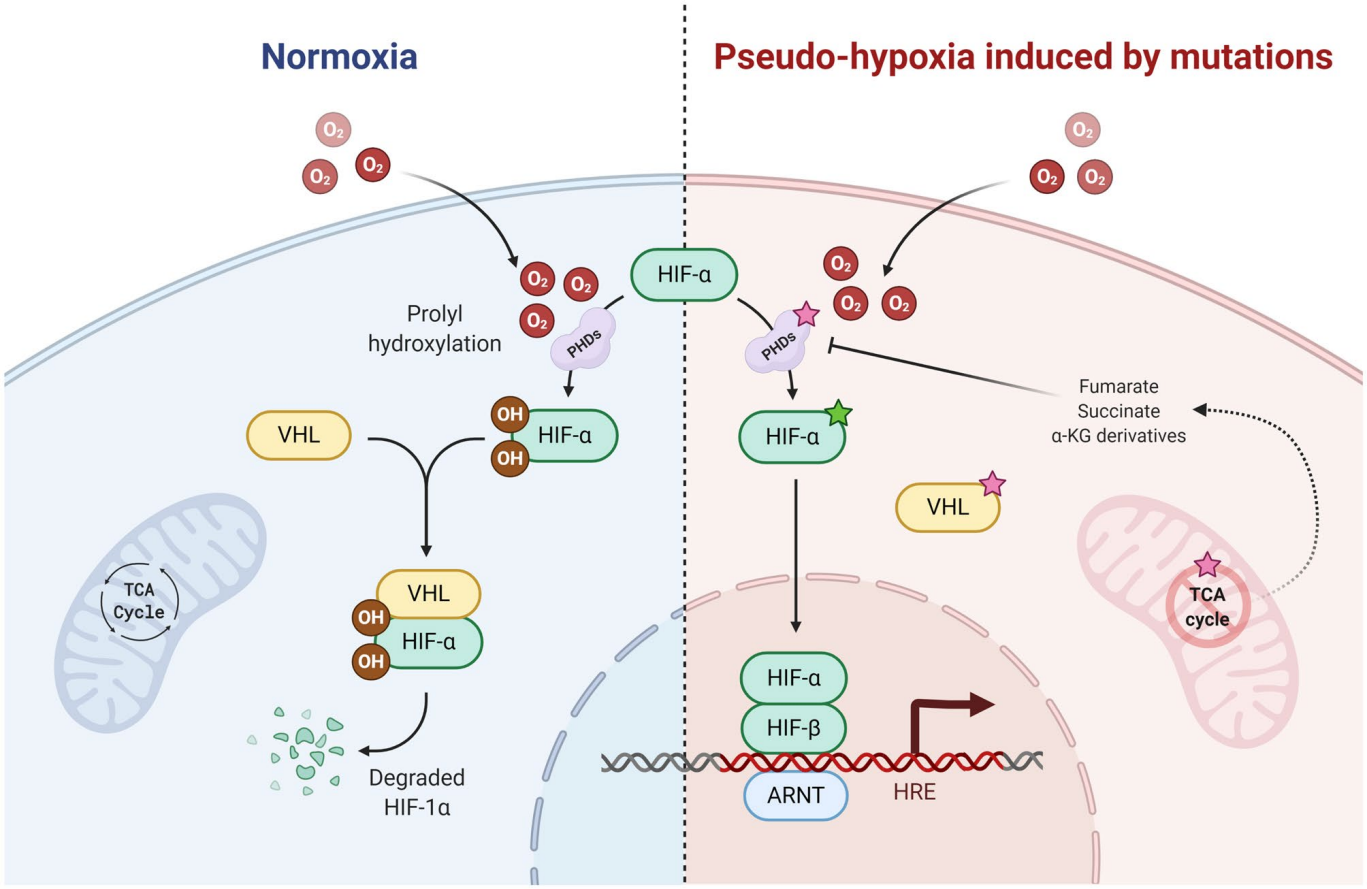
Hypoxia-inducible factor (HIF) pathway regulation in normal states and in PPGL development. In the normal state (left), HIF signaling is impaired by the PHD enzyme class mediated hydroxylation of HIF-α, in turn marking the latter protein for proteolysis via the actions of the von Hippel Lindau (VHL) tumor suppressor protein. If oxygen levels are reduced, HIF-α escapes hydroxylation and degradation, and initiates intra-nuclear target transcriptome programs, including those associated to angiogenesis. In PPGLs (right), activating *EPAS1* (encoding HIF-2α) mutations (green star), inactivating *VHL*, *PHD1/EGLN2* and *PHD2/EGLN1* mutations (red stars) as well as aberrant TCA cycle regulation leading to inhibition of PHDs are main genetic events activating the HIF signaling cascade—even at normal oxygen levels

From a pan-genomic perspective, PPGLs cluster in four main transcriptome groups; the pseudohypoxia cluster, the kinase cluster, the Wingless type (Wnt) cluster and the cortical admixture cluster [29]. The proportion of cases with either adrenal or extra-adrenal localization, the most commonly observed gene mutations, the effects on global methylation levels and risk of metastatic disease for each cluster are illustrated in Fig. 2. Tumors adhering to the kinase cluster (approximately 50% of all PPGLs) are often pheochromocytomas with low metastatic potential, and this cluster contains tumors with either germline or somatic mutations in *NF1*, *RET*, *Myc-associated factor X* (*MAX)*, *Transmembrane Protein 127* (*TMEM127*), *Kinesin Family Member 1B* (*KIF1B-β*), and *HRAS* [29, 38, 41–44]. The pseudohypoxia cluster contains the highest proportion of metastatic PPGLs, and hence, this group of tumors deserve some increased attention [29, 41, 45]. Patients developing tumors that adhere to this cluster are usually young, which is due to an overall high frequency of constitutional mutations in susceptibility genes associated to PPGL development [5]. Interestingly, PPGLs within this cluster can be sub-stratified depending on whether or not gene mutations encoding enzymes responsible for propelling the tricarboxylic acid (TCA) cycle are present (Fig. 3, Fig. 4). TCA cycle aberrant PPGLs in general exhibit the highest risk of metastatic dissemination, which is due to the fact that the accumulation of onco-metabolites will inhibit the cellular effects of dioxygenases, a group of enzymes that catalyze the oxidation of various substrates via the conversion of α-ketoglutarate to succinate (Fig. 5) [46–50]. One important group of inhibited enzymes are the Ten-eleven translocation (TET) proteins responsible for genomic demethylation [49, 51, 52]. When TET enzymes are inhibited, the genome is more prone to hypermethylation of various regulatory regions, of which some have implication for the regulation of tumor development and metastatic spread (Fig. 5) [51–54]. On the other hand, pseudo-hypoxia-driven PPGLs without TCA cycle mutations are generally driven by mutations in the signaling networks that regulate hypoxia-inducible factor (HIF)-mediated transcription of target gene programs (Fig. 6). These events include inactivating mutations of the *VHL*, *Egl-9 Family Hypoxia Inducible Factor 2* (*EGLN2*; encoding prolyl hydroxylase 1, PHD1) and *EGLN1* (encoding PHD2) genes, as well as activating *EPAS1/HIF2-α* mutations [40, 55–57]. The net result is halted degradation of HIF, leading to increased HIF signaling and promotion of angiogenesis and proliferation [57, 58]. However, while TCA cycle aberrant and TCA cycle non-aberrant PPGLs share dysregulation of HIF signaling, the latter group lacks the epigenetic dysregulation caused by TET enzyme inhibition. Given these molecular differences, TCA cycle–driven tumors are even more prone to metastatic spread than other PPGLs within the pseudo-hypoxia cluster, and there is probably a need to distinguish TCA cycle aberrant from TCA cycle non-aberrant PPGLs in terms of risk stratification [5, 59]. This is furthermore mirrored by syndromic manifestations, in which *SDHx* mutated PPGLs display a significant increased risk of disseminated disease, while *VHL* associated tumors rarely exhibit metastatic potential—although adhering to the same transcriptional cluster [5].

The Wnt cluster contains mostly pheochromocytomas with somatic fusions involving the *Mastermind Like Transcriptional Coactivator 3* (*MAML3*) gene as well as *Cold Shock Domain Containing E1* (*CDSE1*) mutations [29]. The *MAML3* protein acts as a transcriptional coactivator of NOTCH pathway associated genes, and *MAML3* fusions and *MAML3* overexpression are recurrent features in various tumor types [60–62]. In PPGL, the fusion partners *Upstream Binding Transcription Factor* (*UBTF*) and *Transcription Factor 4* (*TCF4*) promoter regions, stimulate constitutive overexpression of *MAML3* [29]. The *CDSE1* gene encodes an RNA binding protein involved in translational programming and RNA turnover [63, 64]. In PPGL, *CDSE1* mutations are found on the somatic level and are expected to exhibit loss-of-function properties [29]. From a clinical standpoint, this Wnt expressional cluster also contains PPGLs at risk of metastatic dissemination, probably arguing for tumor DNA screening of *MAML3* gene fusions and *CDSE1* mutations as an efficient way to identify additional high-risk cases that will be negative for other TCA cycle and pseudo-hypoxia-related aberrancies [5, 29].

Finally, the cortical admixture cluster is characterized by pheochromocytomas with *NF1* somatic mutations as well as MEN2-related tumors with *RET* germline mutations. This cluster is enriched for adrenal cortical markers such as *CYP11B1* and *CYP21A2*, which is probably due to the interspersion of adrenal cortical cells [29]. PPGL within this cluster exhibit a very low risk of metastatic spread.

### Pinpointing Metastatic Potential using Histological Algorithms—Is it Possible?

Although not fully recommended by the current WHO classification, histological scoring systems are frequently used in the scientific literature when assessing the metastatic potential of PPGLs [1]. Even though the amount of verifying studies still is limited and the reproducibility debated, the intention of these algorithms is to provide the endocrine pathologist with schemes that might facilitate the identification of cases at risk of future dissemination. However, the limitations of these risk assessment models are mandatory to take into consideration when interpreting the outcome of each individual tumor.

**Fig. 7:**
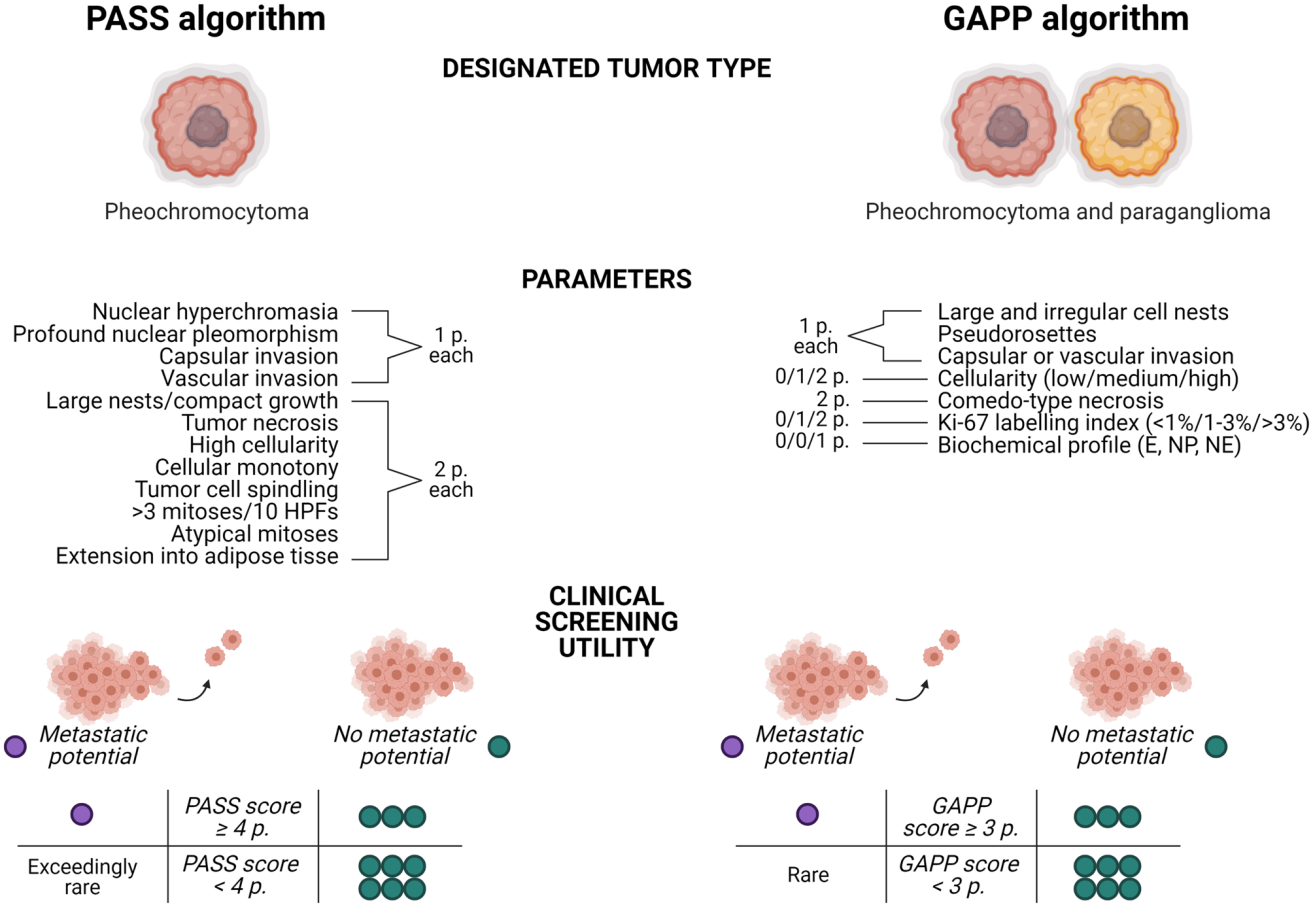
Schematic overview of the Pheochromocytoma of the Adrenal gland Scaled Score (PASS) and the Grading System for Adrenal Pheochromocytoma and Paraganglioma (GAPP) algorithms. Including parameters are listed, as well as the individual points given for each fulfilled criteria. Bottom row depicts the high negative predictive value of both algorithms as suggested by a recent meta-analysis, indicating that low PASS/GAPP scores are strongly associated to benign clinical courses, while elevated PASS/GAPP scores are recurrently reported in metastatic-free PPGLs—thereby limiting the value of these algorithms as “rule-in” tests. P point, E epinephrine, NP non-producing, NE norepinephrine

The Pheochromocytoma of the Adrenal gland Scaled Score (PASS) was developed in 2002 as a method to identify pheochromocytomas with potential for aggressive behavior [65]. Dr. Thompson compared 50 “histologically malignant” and 50 “histologically benign” pheochromocytomas and identified key microscopic features that differed between groups. The algorithm is strictly histology-based and incorporates 12 different parameters that yield a score ranging from 0 to 20 points. One point each is given for the presence of nuclear hyperchromasia, profound nuclear pleomorphism, capsular invasion, or vascular invasion, whereas two points per parameter is given for large nests/compact growth, tumor necrosis, high cellularity, cellular monotony, tumor cell spindling, > 3 mitoses per 10 high power fields, atypical mitoses, and extension into surrounding adipose tisse (Fig. 7). In the original cohort, a PASS score of 4 points or more indicated an increased risk of future aggressive behavior [65]. The scheme has been confirmed in several independent series [66–70], but not in other [71, 72]. Moreover, in terms of intra-observer variability, the PASS algorithm has been proven subpar—with different pathologists reaching different scores for a considerable proportion of cases [72, 73].

**Fig. 8:**
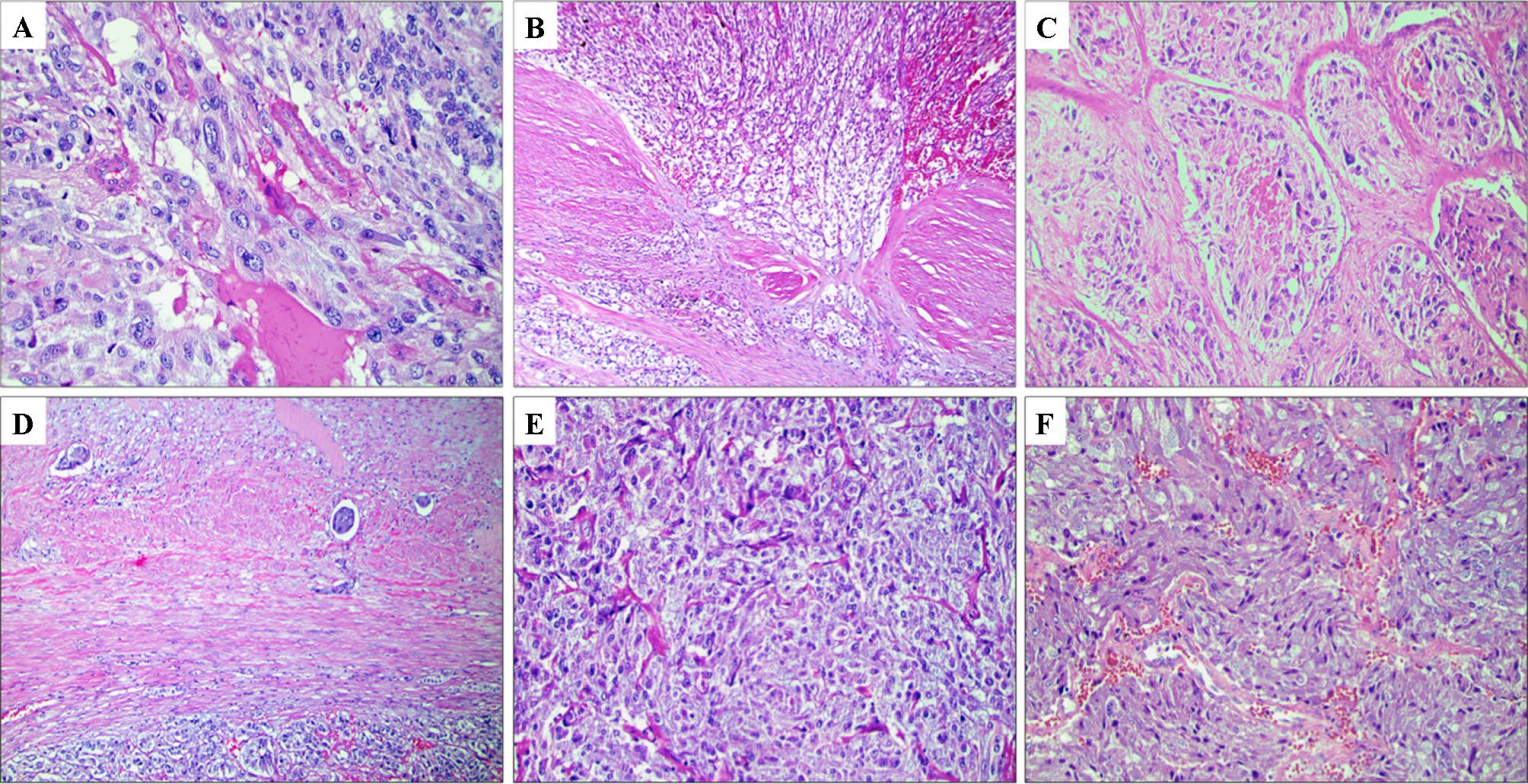
Overstating the risk of aggressive behavior in PPGLs using current risk stratification algorithms. All images are routine hematoxylin–eosin stains. Images **A-D** depict a resected pheochromocytoma with a recurrence-free follow-up time of 20 years. **A** Pleomorphic features. **B** Capsular invasion. **C** Comedo-type necrosis. **D** Vascular invasion. The Pheochromocytoma of the Adrenal gland Scaled Score (PASS) was elevated (5 points); thus, the algorithm falsely identified metastatic potential in this instance. **E**, **F** A large proportion of PPGLs arising in MEN2A patients display large, irregular nests (**E)** and focal tumor cell spindling (**F)**. Large nests is a parameter listed in both the PASS and the Grading System for Adrenal Pheochromocytoma and Paraganglioma (GAPP) algorithms, and tumor cell spindling is an additional PASS related parameter. From a clinical standpoint, pheochromocytomas in MEN2 patients very rarely metastasize

The PASS parameters listed above are classical attributes normally associated to malignant phenotypes in various human tumors. In this aspect, the PASS algorithm could be considered a “histological shotgun,” with a wide spread of microscopic criteria that will pinpoint cases at risk of spread—but also with an increased risk of false positives. Indeed, a recent meta-analysis of > 800 pheochromocytomas with retrievable PASS scores and follow-up data [74] identified this algorithm as highly sensitive for the detection of metastatic cases with an ensuing high negative predictive value. However, the specificity and corresponding positive predictive value were both low (Fig. 7). Therefore, the PASS algorithm could be viewed as a model to rule out metastatic potential (if the score is low), rather than to actually pinpoint cases that will behave malignant. Strikingly, the number of clinically benign pheochromocytomas with elevated PASS scores in the literature surpass the number of reported metastatic cases with similarly high PASS scores [74]. An example of the reduced specificity of the PASS algorithm is provided in Fig. 8, in which a resected pheochromocytoma with a recurrence-free follow-up time of 20 years displayed a pathological PASS score of 5, including vascular invasion, periadrenal invasion, capsular invasion, and nuclear pleomorphism. This serves as an illustration as to how PASS incorrectly may identify pheochromocytomas with little or no metastatic potential as potentially worrisome specimen in need of intensified follow-up. Moreover, specific genotype–phenotype observations of importance for MEN2A-associated PPGL have also been reported, as these tumors often present with large, irregular nests, focal tumor cell spindling, and an elevated Ki-67 index (Fig. 8) [75]. As of this, PPGL patients with germline *RET* mutations may exhibit alarming histological features, although these patients very rarely present with metastatic disease in the clinical setting. Therefore, it is imperative to take into consideration the medical history of each patient when conducting histological assessment of PPGLs—and the abovementioned example also serves to illustrate the importance of molecular genetics in complementing the pathology report in terms of accurate prognostication.

The Grading System for Adrenal Pheochromocytoma and Paraganglioma (GAPP) score was published in 2014 by Dr Kimura and co-workers, in which the authors studied 163 PPGLs, including 40 metastatic cases [76]. Unlike the PASS algorithm, the GAPP study incorporated both pheochromocytomas as well as abdominal paragangliomas, and aimed to highlight contributing factors indicating metastatic potential for this collective tumor group. Building on the PASS algorithm, the GAPP scoring system combines histological findings, immunohistochemistry and clinical information. More specifically, the GAPP score is retrieved by evaluating histological parameters (growth pattern, cellularity, presence of comedo-type necrosis, capsular and vascular invasion), the Ki-67 labelling index as well as the biochemical profile (catecholamine type) (Fig. 7). Noradrenergic PPGLs express low levels of the phenylethanolamine N-methyltransferase (PNMT) enzyme that converts norepinephrine to epinephrine, and reduced PNMT expression is in turn highly related to the pseudo-hypoxia signaling pathway [5, 77]. Therefore, norepinephrine secreting PPGLs are more likely to adhere to transcriptional clusters associated to metastatic behavior, which is also reflected in the GAPP score. The PPGLs are given a score ranging from 0 to 10 points, and subsequently graded as either well differentiated (WD, 0–2 points), moderately differentiated (MD, 3–6 points), or poorly differentiated (PD, 7–10 points). In this study, WD-PPGLs were all metastatic-free, while the metastatic proportion of cases was higher (and the disease-specific survival lower) in the MD and PD groups [76]. Moreover, time to a metastatic event decreased with increased GAPP scores. The GAPP scoring algorithm has only been reproduced in few independent studies and is still a fairly young study in need of additional verification [72, 74, 78]. In the meta-analysis discussed above, the GAPP system exhibited a well-trusted “rule-out” function based on the excellent negative predictive value, similarly to what was shown for PASS (Fig. 7) [74]. However, the rather frequent finding of clinically benign PPGLs with scores ≥ 3 points makes the positive predictive value rather low, and hence puts a strain to the ability to truly identify cases at risk of metastatic spread.

### Early Immunohistochemical Analyses

Given the association between increased mitotic activity and risk of metastatic spread, researches early on turned to the Ki-67 proliferation marker in order to highlight cases at risk of dissemination. Studies seem to agree that clinically aggressive PPGLs are associated to higher Ki-67 indices, although overlaps exist [79–87]. Thus, there seem to be a large amount of scientific data that justified the inclusion of Ki-67 as one key parameter in the GAPP algorithm [76]. As Ki-67 is a part of the antibody lineup in most pathology laboratories and a stain that endocrine pathologists are acquainted to in terms of interpretation, this marker is thus a useful and potentially reproducible tool in the assessment of metastatic potential of PPGLs. Other early observations include the visualization of reduced amounts of sustentacular cells in metastatic PPGLs as visualized via S100 immunohistochemistry [87–92]. However, the S100 staining patterns might be heterogeneous and thereby hard to interpret, especially in larger tumors [89].

### Immunohistochemistry as Molecular Triaging

**Fig. 9:**
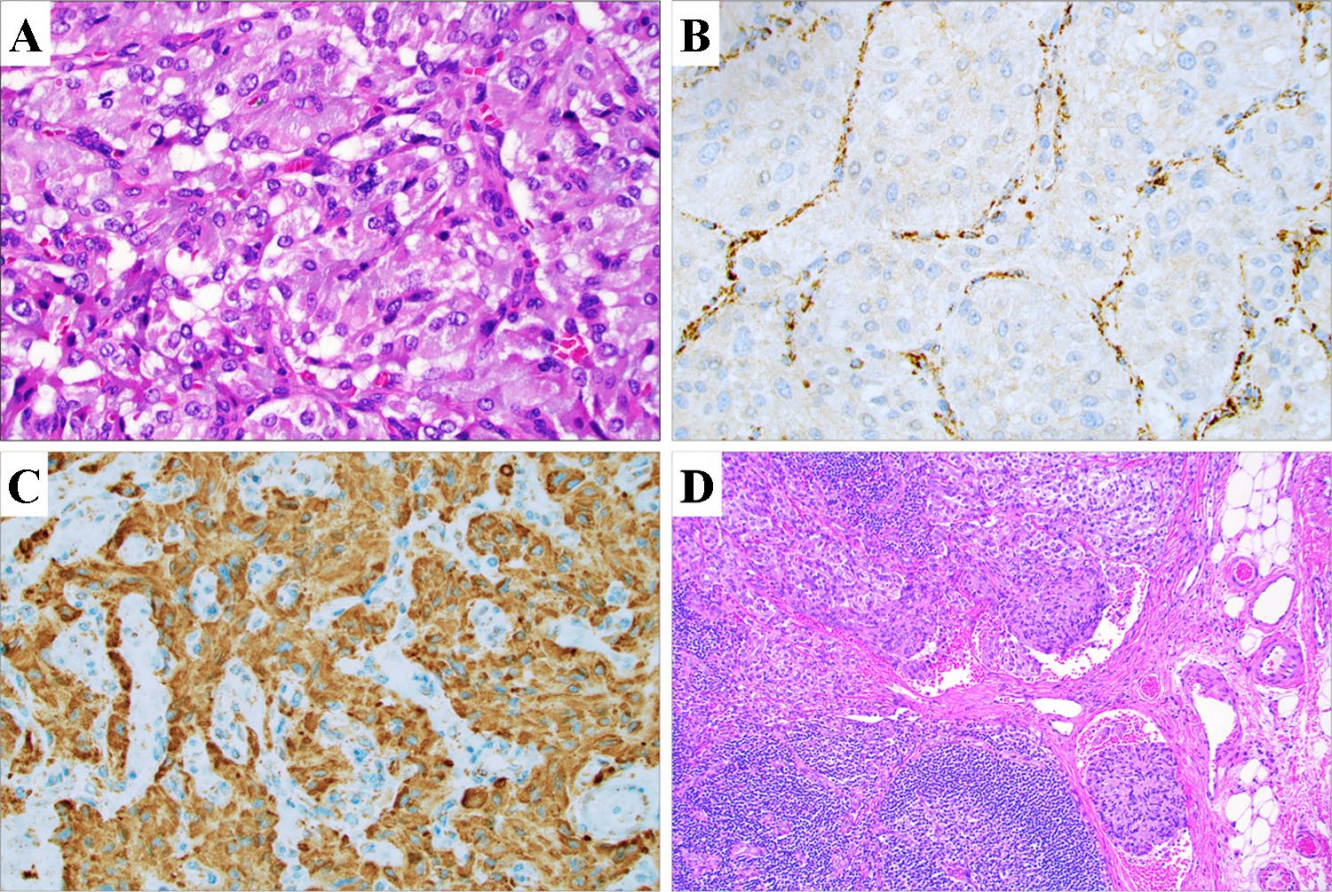
Urinary bladder paraganglioma with an underlying *SDHB* mutation detected through immunohistochemistry. **A** Routine hematoxylin–eosin stain of a urinary bladder paraganglioma arising in a young female patient. **B** SDHB immunohistochemistry revealing absent tumor staining, while adjacent sustentacular cells and stromal components were positive. This staining pattern is highly indicative of an underlying *SDH*x gene mutation. **C** The SDHA immunostaining was positive, as indicated by a granular, cytoplasmic signal. **D** Regional lymph node exhibiting synchronous metastatic deposits. Clinical genetics counseling was initiated, and the patient was found to harbor a germline *SDHB* mutation

The advent of modern next-generation analyses have revolutionized the ability to classify PPGLs, not only in terms of transcriptome clustering but also as a way to detect germline alterations in patients in need of genetic counselling and to pinpoint high-risk mutations in TCA cycle/pseudo-hypoxia-related PPGLs indicating higher risk of metastatic events. Even so, immunohistochemistry is still considered an efficient, cheap, and reproducible method to pinpoint cases in need of intensified molecular studies, as well as to evaluate the functional consequences of some genetic variants of uncertain significance detected through clinical genetics workup [93]. In terms of prognostication and clinical significance, SDHB immunohistochemistry is probably the most well-established marker to date. Following the detection of absent SDHB expression in hereditary PPGLs in patients with germline *SDHB* or *SDHD* mutations, several independent groups have verified the value of SDHB immunohistochemistry to pinpoint SDHx gene mutations occurring either on the somatic or germline level in PPGL [94–97]. The reason behind the ability of SDHB staining to pinpoint cases with either *SDHB*, *C*, or *D* subunit mutations stems from the fact that the succinate dehydrogenase enzyme complex is anchored to the mitochondrial inner membrane via the C and D subunits. Thus, mutational inactivation of *SDHB*, *C*, or *D* will cause a disruption of the entire complex, leading to absent SDHB immunoreactivity (Fig. 9) [98]. The scoring and interpretation of SDHB immunohistochemistry has been proved highly reproducible between pathologists and also a reliable tool in terms of detecting underlying *SDHx* gene mutations [99]. It should however be stressed that subsets of SHDB-immunodeficient PPGLs could display wild-type *SDHx* gene sequences, but instead exhibit *SDHC* promoter hypermethylation, alternatively *VHL* or *NF1* gene mutations [99, 100]. In contrast, *SDHA* mutated PPGLs lose both SDHA and B immunoreactivity, and therefore, SDHA immunohistochemistry could complement the screening panel to detect rare PPGLs with *SDHA* mutations [99, 101]. SDHD immunostaining has also been assessed in PPGLs, in which positive immunoreactivity was observed in *SDHx* gene mutated cases—while wild-type cases stained negative. The reason for this paradoxal and inverted finding could be the potential de-masking of the SDHD epitope upon mutation-mediated disruption of the SDH complex [102]. Moreover, immunohistochemistry targeting fumarate hydratase (FH) has been proven as an efficient method to pinpoint rare *FH* gene germline mutations in PPGL [103], in turn coupled to the hereditary leiomatosis and renal cell carcinoma (HLRCC) syndrome [104].

Apart from the abovementioned markers used to pinpoint PPGLs associated to an aberrant TCA cycle, two markers have shown promise to identify pseudo-hypoxia and kinase cluster associated tumors, respectively, namely carbonic anhydrase IX (CAIX) and MAX. CAIX is frequently found up-regulated in PPGL with underlying *VHL* gene mutations, and the identification of strong immunoreactivity in a PPGL might therefore be a way to identify VHL driven tumors with a lower (but not unneglectable) risk of aggressive behavior than TCA cycle aberrant PPGLs [105]. Similarly, rare cases of *MAX* mutated or gene rearranged PPGLs usually exhibit loss of MAX protein expression—adding yet another potential tool to the diagnostic workup of these lesions [27, 106]. In contrast, NF1 and RET immunohistochemistry cannot yet be recommended as a way to identify underlying *NF1* and *RET* gene mutations, as both sensitivity and specificity have been found reduced in previous studies [107, 108].

Besides immunohistochemical analyses aiding in the context of underlying mutations, an additional marker of prognostic significance include chromogranin B (CHGB), which was the top downregulated gene in an expressional study when stratifying for metastatic PPGLs [70]. The finding was reproduced using immunohistochemistry, with negative or low levels of CHGB immunoreactivity in metastatic PPGLs. Moreover, low preoperative plasma levels of CHGB were associated to higher PASS scores in the resected tumor. Thus, CHGB could possibly act as a preoperative marker of PPGLs with histological worrisome features.

**Fig. 10:**
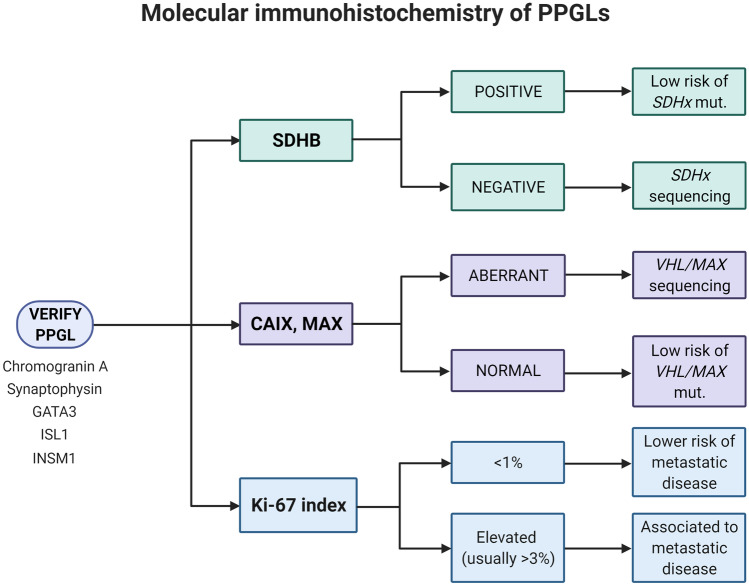
Molecular immunohistochemistry of PPGLs. The endocrine pathologist needs to verify the diagnosis through a concerted action of histology and immunohistochemical markers that pinpoint the chromaffin cell origin. In terms of immunohistochemistry, a succinate dehydrogenase complex flavoprotein subunit B (SDHB) staining could pinpoint cases with underlying *SHDx* gene mutations and an increased risk of disseminated disease. Aberrant carbonic anhydrase IX (CAIX) and MYC associated factor X (MAX) immunostainings might indicate mutations in the *von Hippel Lindau* (*VHL*) or *MAX* genes, respectively, while the proliferation marker Ki-67 could be used to prognosticate the tumors further

Overall, a combined effort of histology and molecular immunohistochemistry is probably needed for the endocrine pathologist in order to better estimate the metastatic potential of each individual PPGL, including diagnostic and prognostic immunohistochemistry (Fig. 10). Moreover, the potential benefit of risk stratification algorithms has to be weighed against the risk of false positives and limited reproducibility, but could potentially be of value to identify cases with little risk of future dissemination.

#### Next-Generation Multi-OMICs Characterization—the Necessary Step

Recent next-generation sequencing studies of PPGLs have increased our understanding of molecular aberrancies that associate to metastatic potential. As previously discussed, gene fusions involving *MAML3* and *CDSE1* mutations are overrepresented in PPGL associated to the Wnt transcriptome cluster, and these tumors have a significantly higher metastatic rate than PPGL associated to the kinase cluster [5, 29]. These aberrancies are somatic and will thus not be identified during a clinical routine screening of germline DNA. Moreover, metastatic PPGLs also harbor somatic gene alterations of potential value for further investigations, such as mutations in transport and cell adhesion genes [109], recurrent *Transcriptional regulator ATRX* (*ATRX*) mutations [29, 110] as well as upregulated *Telomerase reverse transcriptase* (*TERT*) gene expression and *TERT* gene rearrangements [111, 112]. *TERT* gene aberrancies are common in various cancers and usually confer an increased *TERT* mRNA gene output, which is thought to confer immortalization through the elongation of telomeric DNA for these tumor types [85, 112, 113]. *ATRX* encodes a chromatin remodeling protein, and mutations in this gene were intimately coupled to an alternative lengthening of telomeres, furthermore suggesting that the regulation of telomeric regions is crucial for metastatic PPGLs [29]. To add to the association between epigenetic regulators and metastatic potential, somatic mutations in *SET domain containing 2 histone lysine methyltransferase* (*SETD2*) have also been found overrepresented in PPGLs with disseminated disease [29]. *SETD2* is a regulator of various cellular processes such as RNA splicing, DNA repair, DNA methylation and histone modification, and is considered a tumor suppressor in unrelated tumor types [114, 115]. In contrast, mutations in other components of the epigenetic machinery that governs chromatin remodeling are mostly found in metastatic-free PPGLs [31, 116]. Overall, screening for somatic gene aberrancies could complement routine histopathology and molecular immunohistochemical analyses in the hunt for PPGLs with metastatic potential, leading to more efficient pinpointing of high-risk cases.

In terms of epigenetic modifications, we know that especially TCA cycle aberrant PPGLs display unique methylation profiles on both global and gene-specific levels [52, 117, 118]. Although no clear-cut methylation panel for clinical usage in terms of prognostication exists, the association between hypermethylation, TCA cycle defects and metastatic PPGLs could have clinical implications in terms of treatment. For example, as *O(6)-methylguanine-DNA methyltransferase* (*MGMT*) constitutes an epigenetically silenced gene in *SDHx* mutated PPGLs, this could probably explain the partial effect of temozolomide in these cases [119].

## Discussion

PPGLs are enigmatic lesions that pose a serious challenge even to tertiary center experts, not only in terms of clinical handling, but also (and perhaps; particularly) in terms of prognostication of each individual patient. The multifaceted genetic background and the complex association between histological hallmarks of malignancy and clinical evidence of metastatic spread requires a pathologist that can juggle with radiological, biochemical, genetic and histological parameters in order to prognosticate these lesions. Most importantly, an awareness of the limitations of each of these factors to pinpoint risk of disease dissemination is crucial, especially as overconfidence in any parameter might falsely indicate a placid PPGL as potentially aggressive. Thus, a modern endocrine pathologist needs to be updated not only on histological algorithms but also on the ever-dynamic genetic landscape and recent developments within the field of molecular testing. Moreover, additional coordinated multicenter research efforts will most likely be needed to fully dissect the molecular aberrancies that govern the malignant potential of PPGLs.
